# Long-Term Outcomes of Living Kidney Donors Left with Multiple Renal Arteries: A Retrospective Cohort Study from a Single Center

**DOI:** 10.3390/jcm14176121

**Published:** 2025-08-29

**Authors:** Thomas Kurz, Jacob Schmidt, Isabel Lichy, Irena Goranova, Jonathan Jeutner, Nadine Biernath, Lukas Kurz, Thorsten Schlomm, Robert Peters, Frank Friedersdorff, Henning Plage, Bernhard Ralla

**Affiliations:** Charité—Universitätsmedizin Berlin, Corporate Member of Freie Universität Berlin and Humboldt—Universität zu Berlin, Department of Urology, Charitéplatz 1, 10117 Berlin, Germany; thomas.kurz@helios-gesundheit.de (T.K.); till-jacob-valentin.schmidt@charite.de (J.S.); isabel-michaela.lichy@charite.de (I.L.); irena.goranova@charite.de (I.G.); jonathan.jeutner@charite.de (J.J.); nadine.biernath@charite.de (N.B.); lukas.kurz@charite.de (L.K.); thorsten.schlomm@charite.de (T.S.); robert.peters@charite.de (R.P.); frank.friedersdorff@charite.de (F.F.); henning.plage@charite.de (H.P.)

**Keywords:** living kidney donation, multiple renal arteries, donor nephrectomy, renal function, hypertension, vascular anatomy

## Abstract

**Background:** The presence of multiple renal arteries (MRAs) is a common anatomical variant in living kidney donors. While MRAs are not considered a contraindication to donation, it remains uncertain whether leaving the donor with a kidney containing MRAs affects long-term outcomes. This study aimed to evaluate renal and clinical outcomes in donors based on the vascular anatomy of the remnant kidney. **Methods:** We conducted a retrospective cohort study of living kidney donors who underwent nephrectomy at our institution between 2011 and 2016. Donors were categorized according to the vascular anatomy of the remaining kidney: single renal artery (SRA) vs. multiple renal arteries (MRAs). Data on renal function, hypertension, diabetes mellitus, and cardiovascular events were collected at baseline and follow-up. The primary outcome was long-term renal function, which was measured by the estimated glomerular filtration rate (eGFR). Secondary outcomes included clinical comorbidities and postoperative complications. **Results:** Among 190 donors, 132 had a remaining kidney with a single artery and 58 had MRAs. Over a median follow-up of 89.5 months (SRA) and 74.5 months (MRA), there were no significant differences in eGFR (SRA: 66 mL/min vs. MRA: 65 mL/min, *p* = 0.60), serum creatinine (*p* = 0.86), or the incidence of hypertension (31.8% vs. 34.5%, *p* = 0.35). Rates of diabetes mellitus and cardiovascular events were similarly low and comparable between groups. **Conclusions:** Living kidney donors left with a remnant kidney containing multiple renal arteries have similar long-term renal function and clinical outcomes as those with a single renal artery. These findings support the feasibility of MRA retention in donor selection and contribute to evidence-based surgical planning and donor counseling.

## 1. Introduction

Living kidney donation represents the optimal treatment for many patients with end-stage renal disease, providing superior graft survival, reduced waiting times, and better quality of life compared to deceased donor transplantation [1,2]. As donor eligibility criteria broaden to meet increasing demand, anatomical variations—such as the presence of multiple renal arteries (MRAs)—are being encountered more frequently during routine pre-donation imaging with a reported prevalence of up to 30% [3].

The presence of MRAs has historically raised concerns in both donor nephrectomy and transplantation, which is primarily due to potential technical challenges and postoperative complications [4,5]. In recipients, MRAs may be associated with prolonged operative time, increased ischemia, and a higher risk of vascular or ureteral complications [6]. Kok et al. reported that laparoscopic donor nephrectomies involving MRAs were longer in duration with accessory lower pole arteries contributing to a higher rate of ureteral complications [7]. Mahajan et al. similarly noted extended ischemia and operative times in MRA transplants, although one-year graft and patient survival remained comparable to single-artery transplants [8]. Ghazanfar et al. found a modest increase in acute tubular necrosis and vascular complications in recipients of MRA grafts yet no significant differences in long-term outcomes [9]. Overall, these studies support the clinical safety of MRA grafts, contributing to their broader acceptance in transplantation practice.

As the use of MRA grafts becomes routine, a related but less studied question has emerged: does retaining the kidney with multiple arteries in the donor carry any additional long-term risk? Traditionally, donor nephrectomy is guided by split renal function and surgical accessibility, often leaving the donor with the anatomically more complex kidney when both kidneys have similar function. With increased acceptance of complex anatomy in living donor programs, clarifying this risk is important for informed consent and surgical planning.

Although theoretical mechanisms have proposed that MRAs could contribute to segmental perfusion deficits or altered hemodynamics in the remnant kidney, leading to renovascular hypertension, the clinical relevance of this concern remains debated [10]. Some anatomical and imaging studies suggest that MRAs may be associated with a higher prevalence of hypertension or elevated blood pressure. For example, Glodny et al. reported increased plasma renin activity in individuals with MRAs, which could contribute to the development of hypertension [11]. However, other studies have not demonstrated a consistent association. Earlier investigations by Geyer and Poutasse [12], Bönner et al. [13], and Nomura et al. [14] found no significant difference in MRA frequency between hypertensive and normotensive individuals. More recent data from Gandhi et al., based on a large donor cohort, also showed no increased risk of hypertension or renal complications in normotensive donors with MRAs [15]. Taken together, these findings illustrate the uncertainty surrounding the physiological implications of MRAs and highlight the need for more definitive outcome-based research in living donors.

In this study, we sought to evaluate the long-term renal and clinical outcomes of living kidney donors left with a remnant kidney containing multiple renal arteries. By comparing donors with MRAs in the remaining kidney to those with single renal arteries (SRAs), we aimed to clarify whether remnant vascular anatomy is associated with differences in kidney function, hypertension, or other clinical endpoints during long-term follow-up.

## 2. Materials and Methods

### 2.1. Study Design and Population

We performed a retrospective observational cohort study of all living kidney donors who underwent donor nephrectomy at our tertiary academic center between January 2011 and December 2016. Inclusion criteria were as follows: age ≥ 18 years at the time of donation, complete documentation of renal vascular anatomy based on preoperative imaging, and availability of follow-up data for renal function and blood pressure. Donors were categorized into two groups based on the vascular anatomy of the remaining kidney: those with a single renal artery (SRA) and those with multiple renal arteries (MRAs).

### 2.2. Data Collection

Baseline demographic and clinical data were extracted from institutional electronic health records. Renal vascular anatomy was assessed using preoperative contrast-enhanced computed tomography (CT) or magnetic resonance (MR) angiography. Operative and perioperative data were retrieved from surgical reports. The side of donor nephrectomy was determined according to institutional surgical protocol, which prioritizes split renal function. When both kidneys were functionally equivalent, the kidney with a single artery was preferentially selected for removal, and the left kidney was generally favored due to anatomical advantages for transplantation. Postoperative follow-up data were collected from routine outpatient visits, telephone interviews, and general practitioner reports where applicable.

Renal function was assessed using the estimated glomerular filtration rate (eGFR), which was calculated by the CKD–EPI formula. Blood pressure measurements were taken during scheduled follow-ups; hypertension was defined according to the 2018 European Society of Cardiology (ESC) guidelines [16]. Additional comorbidities such as diabetes mellitus, cardiovascular events (myocardial infarction, stroke), and need for renal replacement therapy were recorded. Postoperative complications were classified using the Clavien–Dindo grading system.

### 2.3. Follow-Up and Outcome Measures

The primary outcome was long-term renal function, which was assessed by the eGFR at last follow-up. Secondary outcomes included incidence of arterial hypertension, new-onset diabetes mellitus, cardiovascular events, and need for dialysis. Clinical follow-up was typically conducted at 3 months, 1 year, and then annually.

### 2.4. Statistical Analysis

Continuous variables were tested for normality using the Shapiro–Wilk test. Normally distributed variables were compared between groups using the independent-samples *t*-test, whereas non-normally distributed variables were analyzed with the Mann–Whitney U test. Continuous variables are presented as medians with interquartile ranges (IQRs). Categorical variables were analyzed using the chi-square test or Fisher’s exact test as appropriate. Multivariable analyses were performed to explore the association of group status (SRA vs. MRA) and other confounding variables (age, BMI, gender, operative time, remnant side, preoperative GFR, follow up, and relevant pre-existing conditions) with continuous outcomes (eGFR at last follow-up) using linear regression and with binary outcomes (presence of hypertension during follow-up) using binary logistic regression. A two-sided *p*-value < 0.05 was considered statistically significant. To account for multiple comparisons within each outcome table, *p*-values were adjusted using the Bonferroni correction, and adjusted α-levels were reported accordingly.

Given the smaller size of the MRA group (n = 58) compared to the SRA group (n = 132), a post hoc power analysis was conducted for the two main outcomes: the eGFR at last follow-up and the prevalence of hypertension. The analysis was performed in R using the pwr package. For the eGFR, Cohen’s *d* was calculated, and statistical power and minimal detectable effect size (MDE) were determined. For hypertension, Cohen’s *h* was used to assess power for detecting differences in proportions. Details are provided in Supplementary Table S3.

All statistical analyses were performed using IBM SPSS Statistics for Windows, Version 27.0 (IBM Corp., Armonk, NY, USA) or RStudio 2025.05 (Posit Software, Boston, MA, USA).

## 3. Results

A total of 190 living kidney donors were included in the study: 132 donors had a remnant kidney with a single renal artery (SRA group), and 58 donors had multiple renal arteries (MRA group). Median follow-up was 89.5 months (IQR 39–124) in the SRA group and 74.5 months (IQR 34–116) in the MRA group.

### 3.1. Baseline Characteristics

Baseline demographics and preoperative characteristics were comparable between groups, as shown in Table 1. There were no significant differences in age (SRA: 51.0 ± 10.6 years; MRA: 51.8 ± 10.2 years), sex distribution (SRA: 55.3% female; MRA: 48.3% female), or BMI (SRA: 25.3 ± 3.3 kg/m^2^; MRA: 25.9 ± 3.4 kg/m^2^). Pre-donation kidney function (eGFR) and blood pressure values did not differ significantly.

### 3.2. Distribution of Arterial Anatomy in Extracted Kidneys

The vascular anatomy of the extracted kidneys was analyzed in relation to nephrectomy side and remnant kidney arterial anatomy. In the SRA group, 91 left kidneys (85.8%) and 23 right kidneys (88.5%) had a single artery, while 15 left kidneys (14.2%) and 3 right kidneys (11.5%) had multiple arteries. In the MRA group, 28 left kidneys (66.7%) and 15 right kidneys (93.7%) had a single artery, while 14 left kidneys (33.3%) and 1 right kidney (6.3%) had multiple arteries.

A significant difference was observed in left-sided nephrectomies with extracted kidneys showing a higher rate of multiple arteries in the MRA group compared to the SRA group (33.3% vs. 14.2%, *p* = 0.02). No significant difference was found for right-sided nephrectomies (11.5% vs. 6.3%, *p* = 0.61).

### 3.3. Perioperative Outcomes

Operative parameters including the total operative time, warm ischemia time, and blood loss were similar between the two groups. Intraoperative complications occurred at a low and comparable rate (SRA: 3.1%; MRA: 3.4%; *p* = 0.93). No donor required intraoperative transfusion.

Postoperative complication rates showed some differences: hospital readmissions were more frequent in the MRA group (12.1% vs. 4.5%, *p* = 0.07), and incisional hernias occurred exclusively in MRA donors (3.4% vs. 0%, *p* = 0.03). Postoperative infection was also observed only in MRA donors (5.2%, *p* = 0.008). These infections included two microbiologically confirmed urinary tract infections and one wound infection, which were all occurring in the early postoperative period. No systemic infections or sepsis were recorded. One donor in the MRA group required dialysis during follow-up (Table 2).

### 3.4. Postoperative Medical Conditions During Follow-Up

Long-term follow-up revealed no significant differences in the incidence of arterial hypertension (SRA: 31.8%; MRA: 34.5%, *p* = 0.35, Table 3). Cardiovascular and neurological events were rare and did not differ between groups. No donor in either group developed new-onset diabetes mellitus.

Mild albuminuria was noted in both groups (SRA: 6.1%; MRA: 6.9%), and one case of macroalbuminuria occurred in the SRA group. There were no differences in rates of hematuria, hyperuricemia, or new malignancy. One donor in the SRA group developed renal stones. Infections requiring hospitalization were significantly more frequent in the MRA group (5.2% vs. 0%, *p* = 0.008), but this finding also did not retain significance after correction (adjusted α = 0.0042).

### 3.5. Renal Function and Comorbidities

Median follow-up was 89.5 months in the SRA group and 74.5 months in the MRA group. The eGFR remained stable and comparable between groups at all time points. At final follow-up, the median eGFR was 66 mL/min (SRA) vs. 65 mL/min (MRA), *p* = 0.60.

Hypertension developed in 31.8% of SRA donors and 34.5% of MRA donors (*p* = 0.35). Kaplan–Meier analysis confirmed no significant difference in time to hypertension diagnosis. Rates of diabetes, albuminuria, and cardiovascular disease were also comparable. One MRA donor required dialysis eight years after donation due to ischemic nephropathy in a polar segment. Infections (UTI or pneumonia) were more frequent in the MRA group (5.2%, *p* = 0.008), but no common etiology was identified. In multivariable analyses, linear regression did not identify a statistically significant association between the presence of MRA, age, BMI, gender, operative time, remnant side, preoperative GFR, follow-up, and relevant pre-existing conditions and the eGFR at last follow-up (Supplementary Table S1). Logistic regression showed no statistically significant association between the mentioned parameters and the presence of hypertension during follow-up (Supplementary Table S2).

### 3.6. Postoperative Laboratory Parameters at Follow-Up

Laboratory values obtained at the most recent follow-up showed no clinically relevant differences (Table 4). The median serum creatinine was slightly higher in the MRA group (1.08 mg/dL vs. 1.04 mg/dL), while the eGFR values were similar (MRA: 65 mL/min vs. SRA: 66 mL/min, *p* = 0.60). Hemoglobin and hematocrit values were comparable between groups, as were serum albumin and CRP concentrations. The distribution of CKD stages (mostly CKD G2) was not significantly different.

### 3.7. Post-Hoc Power Analysis

For the eGFR at last follow-up, a Cohen’s d of −0.098 was observed. The calculated power for this difference was 0.10. For hypertension, a Cohen’s h of 0.077 and a calculated power of 0.08 was observed. Based on the current sample sizes, the minimal detectable difference (MDE) for 80% power was 6.4 mL/min/1.73 m^2^ for the eGFR and ±20 percentage points for hypertension (Supplementary Tables S3 and S4).

## 4. Discussion

Our findings confirm that living kidney donors left with a remnant kidney containing MRAs exhibit long-term renal and clinical outcomes comparable to those left with SRAs. Across a median follow-up of over six years, there were no statistically significant differences between groups regarding the eGFR, serum creatinine, incidence of hypertension, or the development of comorbidities.

At final follow-up, the eGFR remained stable and similar in both groups (SRA: 66 mL/min vs. MRA: 65 mL/min, *p* = 0.60), and serum creatinine levels did not differ (*p* = 0.86). Hypertension occurred in 31.8% of SRA and 34.5% of MRA donors (*p* = 0.35) with no significant difference in time to onset in Kaplan–Meier analysis. Similarly, albuminuria rates (SRA: 24.2% vs. MRA: 22.4%) and other metabolic and cardiovascular outcomes were comparable. One MRA donor required dialysis due to localized ischemic nephropathy in a polar segment.

These findings remained robust in multivariate regression analysis adjusting for potential confounding variables, including age, BMI, gender, preoperative GFR, operative time, remnant side, and follow-up duration. MRA status was not significantly associated with either long-term eGFR or the development of hypertension. This supports the notion that vascular anatomy alone does not adversely affect long-term donor outcomes when appropriate donor selection criteria are applied.

Our results align with prior large-scale studies such as Gandhi et al. [15], who examined over 5000 donors and found no association between renal artery anatomy and long-term renal function, hypertension, or cardiovascular events. Similarly, Fehrman-Ekholm et al. [17] and Rizzari et al. [18] reported equivalent donor outcomes in Swedish and North American cohorts, respectively, reinforcing the generalizability of our findings.

Perioperative outcomes were also largely comparable. Although postoperative complications like incisional hernias (3.4%, *p* = 0.03) and infections (5.2%, *p* = 0.008) occurred more frequently in MRA donors, intraoperative complications and overall morbidity were low. These findings are likely multifactorial and not directly attributable to arterial anatomy. Increased vascular complexity may contribute indirectly by prolonging operative time or requiring more extensive dissection. Similar observations were made by Carter et al. [6] and Choi et al. [19], who reported longer warm ischemia times in MRA nephrectomies without associated functional decline.

These observations are consistent with reports by Seet et al. [20], Afshari et al. [21], and Şahin et al. [22], who found that MRAs were associated with increased surgical complexity—such as longer operative or ischemia times—and a slightly higher incidence of early technical complications. However, these did not translate into worse long-term outcomes or increased donor morbidity.

The longstanding concern that MRAs might predispose to segmental hypoperfusion and renovascular hypertension remains largely theoretical. Although systematic perfusion imaging was not performed in our cohort, we found no clinical evidence of functional impairment or elevated risk of hypertension attributable to MRAs. This is consistent with data from Glodny et al. [11], who reported higher plasma renin activity in MRA donors, and Shabalin et al. [23], who found that renovascular hypertension occurred only when one or more arteries were anatomically or functionally compromised. Likewise, large series such as those by Benedetti et al. [24] and Ali-El-Dein et al. [25] found no long-term renal dysfunction in recipients of MRA grafts even when early surgical challenges were encountered.

When stratified by side of nephrectomy, extracted left kidneys showed a significantly higher rate of multiple arteries in the MRA group compared to the SRA group (33.3% vs. 14.2%, *p* = 0.02), whereas no difference was observed in right-sided nephrectomies (*p* = 0.61). This distribution reflects the surgical protocol of selecting the nephrectomy side based primarily on split renal function. When both kidneys are functionally equivalent, the kidney with a single artery is preferentially selected for removal, and the left side is generally favored due to anatomical advantages for transplantation. As a result, donors in the MRA group were more likely to retain kidneys with more complex vascular anatomy without adverse long-term outcomes.

In line with the emerging literature, general anatomical complexity—such as the number or branching pattern of arteries—is not a reliable predictor of long-term function. Specifically, differential renal function and DMSA-based assessments were not associated with the eGFR decline in recent donor cohorts [26]. Instead, studies suggest that remnant cortical volume, as assessed by CT or AI-based tools, may better predict post-donation renal outcomes, especially in older donors [27,28]. Although these methods were not available in our retrospective dataset, they warrant consideration for future prospective studies. Similarly, while early post-donation morphological changes such as compensatory hypertrophy have been described [29], they have not been shown to result in long-term functional decline.

The retrospective design and relatively small sample size, particularly in the MRA group, limit the ability to detect smaller differences or rare events. Importantly, the study was not designed to test equivalence or non-inferiority between groups, and the post hoc power analysis revealed limited statistical power for detecting smaller differences in key outcomes (e.g., 10% for the eGFR and 8% for hypertension). Therefore, while no statistically significant differences were observed, these findings should not be interpreted as evidence of equivalence or comparability between groups. The lack of routine perfusion imaging and reliance on single-center data may also limit generalizability.

Despite these limitations, our findings provide meaningful real-world evidence from a European donor program, supporting current surgical practice that prioritizes split renal function and technical feasibility over arterial anatomy alone. Future multicenter and prospective studies—including perfusion imaging and more granular vascular characterization—are warranted to further refine donor selection and counseling.

## 5. Conclusions

In this retrospective cohort study, we found that living kidney donors left with a remnant kidney containing multiple renal arteries did not show statistically significant differences in long-term renal function or clinical outcomes compared to those left with a kidney with a single artery. No significant differences were observed in key parameters such as the eGFR, serum creatinine, incidence of hypertension, or metabolic and cardiovascular morbidity during long-term follow-up. Although certain postoperative complications were slightly more common in the MRA group, they were not associated with long-term sequelae or diminished renal function.

Our findings support the feasibility of retaining a kidney with multiple renal arteries in the donor when anatomical and functional criteria are otherwise met. These data may help inform surgical decision making and donor counseling in living kidney donation programs, though definitive conclusions about the impact of complex renal arterial anatomy on long-term donor outcomes are limited by the observational nature of the study.

## Figures and Tables

**Table 1:** Demographic and patient characteristics.

Characteristics	SRA (n = 132)	MRA (n = 58)	*p*-Value
Age at transplantation (years)	51.5 (21–58)	52.5 (28–70)	0.75
BMI (kg/m^2^)	26.2 (17.6–36.1)	25.1 (17.9–34.5)	0.30
Remnant side			0.34
Left	26 (19.7%)	15 (25.9%)	
Right	106 (80.3%)	43 (74.1%)	
Number of renal arteries in extracted side			0.02
SRA	114 (86.4%)	42 (72.4%)	
MRA	18 (13.6%)	16 (27.6%)	
Left (single/multiple arteries)	91 (85.8%)/15 (14.2%)	28 (66.7%)/14 (33.3%)	
Right (single/multiple arteries)	23 (83.3%)/3 (16.7%)	15 (93.7%/1 (6.3%)	
Operative time (min)	213 (135–333)	224 (120–392)	0.70
Pre-existing medical condition			
Autoimmune disease	6 (4.5%)	3 (5.2%)	0.85 ^†^
Gynecologic disease	14 (10.6%)	4 (6.9%)	0.16 ^†^
Psychiatric disease	5 (3.8%)	3 (5.2%)	0.66 ^†^
Neurologic disease	4 (3.0%)	3 (5.2%)	0.47 ^†^
Orthopedic disease	2 (1.5%)	0	0.35 ^†^
Gastroenterological disease	12 (9.1%)	6 (10.3%)	0.79 ^†^
Dermatologic disease	0	2 (3.4%)	0.03 ^†^ (ns)
Struma nodosa	3 (2.3%)	1 (1.7%)	0.81 ^†^
Hypothyroidism	12 (9.1%)	3 (5.2%)	0.36 ^†^
Hyperthyroidism	1 (0.8%)	1 (1.7%)	0.55 ^†^
Hyperlipidemia	12 (9.1%)	9 (15.5%)	0.19 ^†^
Diabetes mellitus	3 (2.3%)	0	0.25 ^†^
Adrenal adenoma	2 (1.5%)	0	0.35 ^†^
Hemostasis disorder	8 (6.1%)	3 (5.2%)	0.81 ^†^
Kidney cysts	9 (6.8%)	5 (8.6%)	0.66 ^†^
Bronchial asthma	5 (3.8%)	2 (3.4%)	0.91 ^†^
COPD	1 (0.8%)	0	0.51 ^†^
Cardiologic disease	8 (6.1%)	1 (1.7%)	0.20 ^†^
Arterial hypertension	27 (20.5%)	13 (22.4%)	0.76 ^†^

Values are shown as median (range) or number (percentage of the group); *p*-value < 0.05 in *t*-test, Mann–Whitney U- or Chi-square test.^†^ *p*-values were adjusted for multiple comparisons using the Bonferroni method (adjusted α = 0.0026). Values that were significant before adjustment but not after correction are indicated with “ns” (not significant).

**Table 2:** Perioperative outcome.

Characteristics	SRA (n = 132)	MRA (n = 58)	*p*-Value
Intraoperative complication	4 (3.1%)	2 (3.4%)	0.90
Obstructive vesicoureteral anastomosis	1 (0.8%)	0	
Injury to donor kidney	2 (1.5%)	0	
Splenic lesion	1 (0.8%)	0	
Injury to mesentery of the colon	0	1 (1.7%)	
Conversion due to bleeding	0	1 (1.7%)	
Clavien–Dindo (clinical course)			0.67
II	1 (0.8%)	0	
IIIb	1 (0.8%)	1 (1.7%)	
Hospital readmission	6 (4.5%)	7 (12.1%)	0.06
Reason for readmission			
Hydrocele testis	0	1 (1.7%)	0.13 ^†^
Varicocele testis	0	1 (1.7%)	0.13 ^†^
Incisional hernia	0	2 (3.4%)	0.03 ^†^ (ns)
Inguinal hernia	0	1 (1.7%)	0.13 ^†^
Femoral hernia	0	1 (1.7%)	0.13 ^†^
Epididymitis	1 (0.8%)	1 (1.7%)	0.54 ^†^
Acute kidney injury	1 (0.8%)	0	0.51 ^†^
Postoperative hematoma	1 (0.8%)	1 (1.7%)	0.55 ^†^
Acute postoperative bleeding	0	1 (1.7%)	0.13 ^†^
Pancreatitis	1 (0.8%)	0	0.51 ^†^
Chylous ascites	0	1 (1.7%)	0.13 ^†^
Acute hypertensive crisis	0	1 (1.7%)	0.13 ^†^
Paresthesia	1 (0.8%)	0	0.51 ^†^
LUTS	1 (0.8%)	0	0.51 ^†^

Values are shown as median (range) or number (percentage of the group); *p*-value < 0.05 in Mann–Whitney U-test or Chi-square test.^†^ *p*-values were adjusted for multiple comparisons using the Bonferroni method (adjusted α = 0.0036). Values that were significant before adjustment but not after correction are indicated with “ns” (not significant).

**Table 3:** Postoperative medical condition during follow up.

Characteristics	SRA (n = 132)	MRA (n = 58)	*p*-Value
Follow up (months)	89.5 (5–158)	74.5 (3–163)	0.96
Infection	0	3 (5.2%)	0.008 ^†^ (ns)
Vascular/cardiovascular disease	15 (11.4%)	5 (8.6%)	0.57 ^†^
Malignancy	6 (4.5%)	4 (6.9%)	0.50 ^†^
Neurological/psychiatric disease	10 (7.6%)	3 (5.2%)	0.55 ^†^
Lung disease	3 (2.3%)	1 (1.7%)	0.80 ^†^
Hematological disease/anemia	12 (9.1%)	4 (6.9%)	0.62 ^†^
Incisional hernia or paresthesia/pain	8 (6.1%)	5 (38.5%)	0.52 ^†^
Arterial hypertension (ESC 2018)	42 (31.8%)	20 (34.5%)	0.35
I	30 (23.8%)	19 (33.9%)	
II	5 (4%)	1 (1.8%)	
III	1 (0.8%)	0	
Fatigue symptoms	14 (11.7%)	5 (10%)	0.75 ^†^
Hba1c ≥ 6.5%	18 (13.6%)	3 (5.2%)	0.19 ^†^
Dialysis	0	1 (1.7%)	0.13 ^†^
Albuminuria	32 (24.2%)	13 (22.4%)	0.64 ^†^
Hyperlipidemia	30 (22.7%)	16 (27.6%)	0.47 ^†^

Values are shown as median (range) or number (percentage of the group); *p*-value < 0.05 in Mann–Whitney U-test or Chi-square test.^†^ *p*-values were adjusted for multiple comparisons using the Bonferroni method (adjusted α = 0.0042). Values that were significant before adjustment but not after correction are indicated with “ns” (not significant).

**Table 4:** Kidney function during follow up.

Characteristics	SRA (n = 132)	MRA (n = 58)	*p*-Value
Follow up (months)	89.5 (5–158)	74.5 (3–163)	0.96
Glomerular filtration rate (GFR) (mL/min)			
Preoperative (DTPA)	119 (85–235)	124.2 (76–237)	0.23
At discharge (eGFR)	57 (38–90)	65 (42–90)	0.98 ^†^
1 month (eGFR)	61 (38–97)	62 (44–90)	0.61 ^†^
6 months (eGFR)	62 (38–92)	58.5 (43–84)	0.07 ^†^
1 year (eGFR)	62.5 (37–124)	59 (45–90)	0.96 ^†^
2 years (eGFR)	66 (39–90)	64 (48–90)	0.59 ^†^
3 years (eGFR)	64 (38–90)	66 (49–90)	0.27 ^†^
4 years (eGFR)	64 (36–90)	63 (42–90)	0.96 ^†^
5 years (eGFR)	66 (39–95)	65 (53–90)	0.36 ^†^
At follow up (eGFR)	66 (38–113)	65 (42–90)	0.55 ^†^
Serum creatinine (mg/dL)			
Preoperative	0.78 (0.49–1.17)	0.77 (0.58–1.37)	0.68 ^†^
At discharge	1.25 (0.64–1.77)	1.16 (0.76–2.01)	0.46 ^†^
At follow up	1.05 (0.66–1.80)	1.03 (0.67–1.67)	0.67 ^†^
CKD at follow up			0.09
I (eGFR 60–89 mL/min)	57 (33.1%)	37 (21.5%)	
II (eGFR 45–59 mL/min)	36 (20.9%)	17 (9.9%)	
III (eGFR 30–44 mL/min)	9 (5.2%)	0	

Values are shown as median (range) or number (percentage of the group); *p*-value < 0.05 in *t*-test, Mann–Whitney U- or Chi-square test.^†^ *p*-values were adjusted for multiple comparisons using the Bonferroni method (adjusted α = 0.0038).

## Data Availability

The data presented in this study are available from the corresponding author upon reasonable request. Due to ethical considerations and institutional policies regarding donor privacy and data protection, individual-level clinical data cannot be made publicly available.

## Supplementary Materials

The following supporting information can be downloaded at https://www.mdpi.com/article/10.3390/jcm14176121/s1, Table S1: Multivariable Linear Regression Analysis of Factors Associated with eGFR at Last Follow-up; Table S2: Multivariable Logistic Regression Analysis of Factors Associated with Incident; Table S3: Post-hoc Power Analysis; Table S4: Power and Minimal Detectable Effects (MDE).

## Abbreviations

The following abbreviations are used in this manuscript:

MRAs	Multiple Renal Arteries
SRA	Single Renal Artery
eGFR	Estimated Glomerular Filtration Rate
CKD-EPI	Chronic Kidney Disease Epidemiology Collaboration
CT	Computed Tomography
MR	Magnetic Resonance
ESC	European Society of Cardiology
RRT	Renal Replacement Therapy
SD	Standard Deviation
IQR	Interquartile Range
BMI	Body Mass Index
ASA	American Society of Anesthesiologists
DM	Diabetes Mellitus
CVD	Cardiovascular Disease
HTN	Hypertension
SPSS	Statistical Package for the Social Sciences
