# Supplementary material for: Long-Term Outcomes of Living Kidney Donors Left with Multiple Renal Arteries: A Retrospective Cohort Study from a Single Center
Source: J Clin Med. 2025 Aug 29;14(17):6121. doi: 10.3390/jcm14176121 (PMC12429414; doi:10.3390/jcm14176121)
Supplement: Supplementary file 1 [file jcm-14-06121-s001.zip › jcm-3783328-supplementary.pdf]

Table S1. Multivariable Linear Regression Analysis of Factors Associated with eGFR at Last Follow-up; p-value < 0.05 in linear regression analysis.

Characteristics	β (mL/min/1.73 m²)	CI 95%	p-value
MRA in situ: SRA (Ref.) vs. MRA	−1.74	−5.80 to 2.31	0.38
Age at transplantation (years)	−0.61	−0.81 to -0.43	**<0.001**
BMI (kg/m²)	−0.71	−1.26 to −0.15	**0.01**
Gender: female (Ref.) vs. male	1.61	−2.43 to 5.65	0.43
Remnant side: left (Ref.) vs. right	1.35	−3.27 to 5.98	0.58
Operative time (min)	0.03	−0.02 to 0.08	0.28
Preoperative GFR (mL/min/1.73 m²)	0.04	−0.03 to 0.11	0.24
Follow up (months)	0.04	−0.01 to 0.08	0.11
Pre-existing Diabetes mellitus: no (Ref.) vs. yes	−11.15	−29.04 to 6.74	0.22
Pre-existing Kidney cysts: no (Ref.) vs. yes	−0.13	−7.46 to 7.20	0.97
Pre-existing Cardiologic disease: no (Ref.) vs. yes	−0.30	−9.10 to 8.50	0.95
Pre-existing Arterial hypertension: no (Ref.) vs. yes	−3.84	−8.99 to 1.30	0.14

Abbreviations: SRA: single renal artery; MRA: multiple renal arteries; BMI: Body Mass Index; CI: confidence interval

Table S2 Multivariable Logistic Regression Analysis of Factors Associated with Incident Hypertension During Follow-up; p-value < 0.05 in logistic regression analysis.

Characteristics	Adjusted Odds Ratio	CI 95%	p-value
MRA in situ: SRA (Ref.) vs. MRA	0.42	0.16–1.07	0.07
Age at transplantation (years)	1.01	0.97–1.05	0.86
BMI (kg/m²)	1.01	0.91–1.13	0.83
Gender: female (Ref.) vs. male	1.05	0.46–2.42	0.90
Remnant side: left (Ref.) vs. right	0.65	0.27–1.57	0.34
Operative time (min)	0.99	0.99–1.01	0.62
Preoperative GFR (mL/min/1.73 m²)	1.01	0.99 to 1.02	0.52
Follow up (months)	0.99	0.99–1.01	0.71
Pre-existing Diabetes mellitus: no (Ref.) vs. yes	0		1
Pre-existing Kidney cysts: no (Ref.) vs. yes	1.57	0.42–5.87	0.50
Pre-existing Cardiologic disease: no (Ref.) vs. yes	1.11	0.20–6.24	0.91
Pre-existing Arterial hypertension: no (Ref.) vs. yes	0.77	0.25–2.30	0.63

Abbreviations: SRA: single renal artery; MRA: multiple renal arteries; BMI: Body Mass Index; CI: confidence interval

Table S3 Post-hoc Power Analysis.

Outcome	n (MRA)	n (SRA)	MRA Mean ± SD / %	SRA Mean ± SD / %
eGFR (mL/min/1.73 m²)	58	132	66.2 ± 12.2	67.6 ± 15.3
Hypertension (%)	58	132	35.7%	32.1%

Table S4 Power and Minimal Detectable Effects (MDE).

Outcome	Effect size	Achieved power (α = 0.05)	MDE (80% power)
eGFR (continuous)	Cohen's d = −0.098	0.10	Δ_min ≈ 6.41 mL/min/1.73 m^2 (d_min = 0.444)
Hypertension (binary)	Cohen's h = 0.077	0.08	↑ +21.7 %− pts (RR_min ≈ 1.68) ↓ −18.2 %− pts (RR_min ≈ 0.43)
